# Principles of Food Safety and Toxicological Risk Assessment: Refining Hazards, Risk Management Communication, and Common Misconceptions

**DOI:** 10.1111/1541-4337.70552

**Published:** 2026-07-09

**Authors:** Kenneth S. Rivera‐González, Jay S. Petrick, Christine M. Crincoli, Matthew D. Teegarden, Paul R. Hanlon

**Affiliations:** ^1^ Abbott Nutrition Abbott Park Illinois USA; ^2^ Archer Daniels Midland Company Decatur Illinois USA; ^3^ Cargill, Inc. Mayzata Minnesota USA

## Abstract

Toxicology has evolved into a robust scientific discipline since the 16th century when Paracelsus, the “Father of Toxicology,” established the basic yet fundamental principle that “the dose makes the poison.” Toxicology is often first associated with the chemical and pharmaceutical industries; however, it plays an equally relevant role in evaluating food chemical safety, a field gaining increasing attention from both the scientific community and consumers. Though essential for human survival, foods can also pose risks, underscoring the relevance of the toxicological principle of “the dose makes the poison.” The fundamental principles of toxicology are often misunderstood, miscommunicated, and misperceived in the context of food safety risk assessments. Importantly, this misalignment among scientists and science communicators can promulgate further issues of false information, confusion, and distrust with consumers. This paper presents key terminology and concepts used in food toxicology with the ultimate goal of promoting an accurate and consistent understanding among scientists and communicators within the broad field of food science.

**Abbreviations**
ADI: acceptable daily intake
ALARA: as low as reasonably achievable
ALOP: appropriate level of protection
ARfD: acute reference dose
BMD: benchmark dose
BMDL: benchmark dose level
CFR: Code of Federal Regulations
EDI: estimated daily intake
EDT: expanded decision tree
EFSA: European Food Safety Authority
EHC 240: Environmental Health Criteria 240
FAO: Food and Agricultural Organization
GMP: Good Manufacturing Practices
HBGV: health‐based guidance value
IARC: International Agency for Research on Cancer
JECFA: Joint Expert Committee on Food Additives
LB: lower bound
LD_50_
: median lethal‐dose
LOAEL: lowest observed adverse effect level
LOD: limit of detection
LOEL: lowest observed effect level
LOQ: limit of quantification
MADL: maximum allowable dose level
MB: medium bound
ML: maximum limit
MOE: margin of exposure
MOS: margin of safety
MRL: maximum residue limit
NAM: new approach methodology
NAS: National Academy of Sciences
NIH: National Institute of Health
NOAEL: no observable adverse effect level
NOEL: no observed effect level
NRC: National Research Council
OECD: Organisation for Economic Co‐operation and Development
OEHHA: Office of Environmental Health Hazard Assessment
POD: point of departure
PTWI: provisional tolerable weekly intake
QSAR: quantitative structure-activity relationship
RfD: reference dose
TDI: tolerable daily intake
TTC: threshold of toxicological concern
UB: upper bound
UL: upper tolerable intake limit
US EPA: US Environmental Protection Agency
US FDA: United States Food and Drug Administration
WHO: World Health Organization

## 1 Introduction

From the foundational perspectives laid out in *Casarett and Doull's Toxicology: The Basic Science of Poisons* to the discussion in recent texts such as *Goodman and Gilman's The Pharmacological Basis of Therapeutics*, there is consistent recognition that defining a poison remains challenging, a view reflected in the statement that “Toxicology is often regarded as the science of poisons or poisoning, but developing a strict definition for poison is problematic” (Casarett and Doull 1975; Brunton et al. 2011; Grandjean 2016).

The reason for this paradox is that, in principle, any substance, from essential nutrients to drugs, has the capacity to harm a living organism and, therefore, act like a poison. This discovery is credited to the famous Renaissance physician Paracelsus (1493–1541), often referred to as the “Father of Toxicology.” Writing primarily in German, his key statement can be translated as: “What is there that is not poison? All things are poison, and nothing is without poison. Solely the dose determines that a thing is not a poison.” Paracelsus paved the way for modern toxicologists to develop the concept of thresholds, which defined the no observable adverse effect level (NOAEL) as the highest tested dose or concentration of a substance at which no adverse effects are observed. Essentially, by demonstrating that lower doses will reduce and ultimately eliminate the occurrence of adverse effects, Paracelsus contributed to the modern distinction between hazard and risk, now simply expressed by the phrase “the dose makes the poison.”

Centuries after Paracelsus, our understanding of the impact of chemicals on human health has greatly expanded the application of toxicology to practically all industries, including the food industry. Fields such as occupational safety and drug development have progressed in establishing the use of toxicology to drive public policy. Despite this progress in other fields, there is still significant policy debate concerning food chemical risk assessment in terms of what it means for food to be “safe.” Much of what appears to be impeding progress in food policy does not stem from scientific uncertainty but rather from differences in public perception and policy, particularly the misconception that “safe” implies zero risk, which is unrealistic for chemicals. This is in contrast to microbiological food safety risk assessments and management procedures. Microbial agents have the ability to exponentially grow, thereby increasing risk even from low initial levels of contamination; thus, the presence of a pathogen is already sufficient to drive the risk management decision toward a more hazard‐based approach. Chemical contamination, on the other hand, is typically managed using a risk based‐approach with dose–response relationships and established exposure thresholds rather than presence alone. Ultimately, determining what is considered to be “sufficiently safe” is a matter of policy judgement.

Public policy managing exposure to chemicals has always presented a challenge to government agencies. In 1983, the United States National Academy of Sciences (NAS) published *Risk Assessment in the Federal Government: Managing the Process*. The report sets the tone by stating that:

This report explores the intricate relations between science and policy in a field that is the subject of much debate—the assessment of the risk of cancer and other adverse health effects associated with exposure of humans to toxic substances. (National Research Council [US] Committee on the Institutional Means for Assessment of Risks to Public Health 1983)

And continues to highlight the controversy that exists in setting public health policy with:

Many decisions of federal agencies in regulating chronic health hazards have been bitterly controversial. The roots of the controversy lie in improvements in scientific and technologic capability to detect potentially hazardous chemicals, in changes in public expectations and concerns about health protection… (National Research Council (US) Committee on the Institutional Means for Assessment of Risks to Public Health 1983)

Over four decades later, this general debate still exists, and concern about food being a source of “toxic substances” has only increased. These concerns are often amplified by the media, influencers, and other outlets that might not fully grasp the complexity of toxicological principles. As a result, various forms of false information can spread quickly, further affecting the public perception of risk. *Misinformation* refers to the unintentional spread of inaccurate information (e.g., claiming that apples are unsafe due to the presence of cyanide in their seeds), while *disinformation* involves the deliberate sharing of falsehoods to mislead or manipulate (e.g., claiming that all processed foods are unhealthy). *Malinformation*, on the other hand, occurs when accurate information is taken out of context to promote biased or misleading narratives (e.g., intentionally excluding exposure context to claim a product as hazardous based solely on the presence of a detectable chemical). Collectively, these forms of false information can be detrimental to the consumers' trust in food safety systems and can lead to unnecessary avoidance of nutritious foods (Quam and Casavale 2017; Liu and Ralston 2021).

It is critical to expand our understanding of the substances humans can be exposed to through food in order to drive science‐based policies and other decision‐making. However, it is also necessary to evaluate the presence of these substances in the proper context: Why are they there? At what levels? The route of exposure from which toxicological data are generated is also critical to consider. Food safety assessment should prioritize data from studies where a substance is consumed orally rather than extrapolating studies where the substance is either inhaled or absorbed through the skin (dermal). Toxicity claims regarding the components of food without the proper context of exposure can be misleading and compromise informed consumer decisions.

An added complication with food safety risk assessment, as opposed to fields such as pharmaceuticals, is the need to disentangle the effects caused by the general diet itself from the toxicity of one dietary component. Unlike drugs, which are usually taken in controlled doses for specific purposes during a determined period of time, foods are consumed in combination with many other substances over a long period of time, sometimes even a lifetime. This makes it difficult to determine whether an observed adverse effect after consuming a food is caused by a particular ingredient, the broader diet, or interactions between multiple dietary components. Except for carefully controlled laboratory studies, it can be extremely challenging to definitively determine whether an observed effect is due to the presence or absence of a substance in the diet or due to a complicated interaction between both factors.

Food safety risk assessment is a nuanced subject in societies where what we eat can be sacred, personal, cultural, and integral to our identity. Thus, objectivity can be hard to maintain when assessing and communicating risk. The goal of food toxicology is to provide accurate, science‐based information so that all stakeholders, including manufacturers, consumers, and regulators, can make informed decisions. However, there is a lack of a common understanding of food safety risk assessment terms, as illustrated by the variability in regulatory definitions outlined in Table 1. This variability can create confusion for stakeholders that might be unfamiliar with these differences when discussing how to use risk assessment outcomes to guide food decision‐making.

**TABLE 1:** Comparative regulatory definitions of core food safety terms across the FDA, EFSA, and Codex.

Term	Regulatory body definition		
	US (FDA)	EU Commission	Codex Alimentarius
Food additive	“any substance the intended use of which results or may reasonably be expected to result, directly or indirectly, in it becoming a component or otherwise affecting the characteristic of any food.” (U.S. Food and Drug Administration 2018)	“substances that are not normally consumed as food itself but are added to food intentionally for a technological purpose.” (European Parliament and Council 2008)	“any substance not normally consumed as a food by itself and not normally used as a typical ingredient of the food, whether or not it has nutritive value, the intentional addition of which to food for a technological (including organoleptic) purpose in the manufacture, processing, preparation, treatment, packing, packaging, transport or holding of such food results, or may be reasonably expected to result (directly or indirectly) in it or its by‐products becoming a component of or otherwise affecting the characteristics of such foods.” (Codex Alimentarius Commission 2026c)
Processing aid	Substances that are added during the processing of food but are: “removed in some manner from the food before it is packaged in its finished form, or are converted into constituents normally present in the food, or are present in the finished product at insignificant levels” (U.S. Food and Drug Administration 1977b).	Any substance which: is not consumed as a food by itself; is intentionally used in the processing of raw materials, foods or their ingredients, to fulfil a certain technological purpose during treatment or processing; and may result in the unintentional but technically unavoidable presence in the final product of residues of the substance or its derivatives provided they do not present any health risk and do not have any technological effect on the final product (European Parliament and Council 2008).	“a substance or material not including apparatus or utensils and not consumed as a food ingredient by itself, intentionally used in the processing of raw materials, foods or its ingredients to fulfil a certain technological purpose during treatment or processing and which may result in the non‐intentional but unavoidable presence of residues or derivatives in the final product.” (Codex Standard 107‐1981 1981)
Contaminant	“include a broad range of chemicals that may be present in food and that have the potential to cause harm.” (U.S. Food and Drug Administration 2005)	“any substance not intentionally added to food which is present in such food as a result of the production (including operations carried out in crop husbandry, animal husbandry and veterinary medicine), manufacture, processing, preparation, treatment, packing, packaging, transport or holding of such food, or as a result of environmental contamination. Extraneous matter, such as, for example, insect fragments, animal hair, etc, is not covered by this definition.” (Council of the European Communities 1993)	“any substance not intentionally added to food or feed for food producing animals, which is present in such food or feed as a result of the production (including operations carried out in crop husbandry, animal husbandry and veterinary medicine), manufacture, processing, preparation, treatment, packing, packaging, transport or holding of such food or feed, or as a result of environmental contamination. The term does not include insect fragments, rodent hairs and other extraneous matter.” (Codex Alimentarius Commission 2026a)
Pesticide	“any substance or mixture of substances intended for preventing, destroying, repelling, or mitigating any pest.” (U.S. Environmental Protection Agency 1996)	“‘pesticide residues’ means residues, including active substances, metabolites and/or breakdown or reaction products of active substances currently or formerly used in plant protection products as defined in Article 2, point 1 of Directive 91/414/EEC, … including in particular those which may arise as a result of use in plant protection, in veterinary medicine and as a biocide.” (European Parliament and Council 2005)	“any substance intended for preventing, destroying, attracting, repelling, or controlling any pest including unwanted species of plants or animals during the production, storage, transport, distribution, and processing of food, agricultural commodities, or animal feeds or which may be administered to animals for the control of ectoparasites.” (Codex Alimentarius Commission 2026c)
Color additive	“any dye, pigment, or other substance which when added or applied to a food, drug, cosmetic, or to the human body, is capable (alone or through reactions with other substances) of imparting color” (U.S. Food and Drug Administration 2025a).	Additives added to food for: “restoring the original appearance of food of which the colour has been affected by processing, storage, packaging and distribution, whereby visual acceptability may have been impaired; making food more visually appealing; giving colour to food otherwise colourless.” (European Parliament and Council 2008).	“A food additive, which adds or restores color in a food.” (Codex Alimentarius Commission 2026b)
Novel food	“Innovations in food ingredients and food production technologies provide consumers additional food choices … Some of these ingredients and foods are new to the supply chain, and additional scientific information related to these new food ingredients is valuable” (U.S. Food and Drug Administration 2026).	“food that had not been consumed to a significant degree by humans in the EU before 15 May 1997, when the first Regulation on novel food came into force. ‘Novel Food' can be newly developed, innovative food, food produced using new technologies and production processes, as well as food which is or has been traditionally eaten outside of the EU.” (European Commission 2026)	N/A
Food additive identifiers	The US FDA does not maintain a numerical ID system for food additives, instead these are listed under the Food Additive regulation (21 CFR Part 170).	E Number: “A number used in the European Union to identify permitted food additives. An E number means that an additive has passed safety tests and has been approved for use.” (European Food Safety Authority 2026)	The Codex International Numbering System (INS) “is intended as a harmonized naming system for food additives as an alternative to the use of the specific name, which may be lengthy. Inclusion in the INS does not imply approval by Codex for use as food additives” (Codex Alimentarius Commission 2025).
Terms prescribing the conditions for the safe use of food additives	Good Manufacturing Practices (GMP) restrictions include: “The quantity of the substance added to food does not exceed the amount reasonably required to accomplish its intended physical, nutritive, or other technical effect in food. Any substance intended for use in or on food is of appropriate food grade and is prepared and handled as a food ingredient.” (U.S. Food and Drug Administration 1977a)	Quantum Satis (Q.S.): “It means that no maximum numerical level is specified, and substances shall be used in accordance with good manufacturing practice, at a level not higher than is necessary to achieve the intended purpose and provided the consumer is not misled.” (European Commission 2021)	Good Manufacturing Practices (GMP) for food additives include: “the quantity of the additive added to food shall be limited to the lowest possible level necessary to accomplish its desired effect; the quantity of the additive that becomes a component of food as a result of its use in the manufacturing, processing or packaging of a food and which is not intended to accomplish any physical, or other technical effect in the food itself, is reduced to the extent reasonably possible; and, the additive is prepared and handled in the same way as a food ingredient.” (Codex Alimentarius Commission 2026c)

This paper seeks to define toxicological terms and concepts in the context of food chemical safety risk assessments of whole foods, food components, and unintended substances, as well as food ingredients in an effort to drive consistency in discussions across all decision‑making entities. The intention is to establish a common language to aid in the harmonization of food chemical risk assessments and provide more consistency and effectiveness in the communication of risk assessment outcomes.

## 2 Terminology Used to Describe and Categorize Food Substances

Foods contain numerous substances with diverse chemical properties, the levels and bioavailability of which can be affected by numerous factors including but not limited to geographic origin, extent of processing, and product formulation. What is common for all of these substances is that, as Paracelsus established, at a high enough dose, they can be toxic to humans, and at a low enough dose, they are mostly non‐toxic, as the lowest dose level eliciting adverse effects depends on the intrinsic toxicity of each substance. Despite this, many different terms are used for food substances, and the use of multiple terms could incorrectly lead individuals to believe that a substance poses a risk merely because of the nomenclature used.

A *chemical* refers to any discrete substance, whether it occurs naturally, is isolated from a natural source, or is synthetically produced. It is also recognized that certain chemical mixtures are treated as discrete substances for regulatory or practical purposes, including galacto‐oligosaccharides and starch hydrolysates, which consist of molecules with varying chain lengths. In risk assessment, chemical and *substance* are often used interchangeably, with no intended distinction between the two terms. Some consumer‐facing publications use the term “chemical” as a shorthand for “chemically‐synthesized”; however, the origin (e.g., naturally occurring vs. chemically synthesized) of a substance does not impact the risk assessment of that substance. For example, isoamyl acetate is a chemical responsible for the characteristic flavor and aroma of bananas. Whether isoamyl acetate is naturally present in a banana or chemically synthesized for the purpose of flavoring foods, it is still the same chemical. Chemically synthesized and naturally occurring isoamyl acetate are identical in chemical structure and physiochemical properties and therefore share the same toxicological properties.

As we advance the capabilities of analytical methodology, the list of chemicals known to be present in foods has expanded significantly and now includes numerous substances that have been present in our diets, undetected and/or uncharacterized, for millennia. The misleading classification of substances as chemicals (or chemically synthesized) can create misconceptions about their safety and nutritional value due to the negative connotation some consumers relate to artificiality, even if some of these chemicals have been in our food for as long as we have been consuming it. Additionally, the technical name of a substance does not directly relate to the safety of the substance as all discrete substances can be called by their technical name, including the water we drink and the air we breathe.

Some substances are referred to as being *inherent* to food. This term is used to describe a substance that is part of a food or food ingredient and is permanent and/or inseparable from it. These substances may or may not be able to be removed without altering the original properties of the food. For example, caffeine is a substance that is inherent in coffee beans and tea leaves and can be removed through processing to produce decaffeinated products.

*Allergens* are another group of substances present in some foods that are naturally occurring, otherwise harmless proteins capable of triggering an immune‐mediated reaction in sensitive individuals (Codex Alimentarius Commision 2020). Allergens can cause immune‐mediated reactions ranging from mild gastrointestinal discomfort and skin rashes to potentially life‐threatening side effects like swelling of the airways, depending on the level of allergen exposure and the individual threshold to elicit a response. It is important to note that not all adverse reactions to food are food allergies. Some reactions, such as those experienced by individuals with lactose or gluten intolerances, are due to food intolerances, which are not immune‐mediated (Taylor and Baumert 2020).

There are also different terms used to categorize different types of substances intentionally added to food. In the United States, the term *food additive* broadly refers to any substance that could be reasonably expected to be found in food or that could affect the characteristics of the food (U.S. Food and Drug Administration 1977b). Internationally, such as in Europe and in the standards established by Codex Alimentarius, the term food additive is used only for substances that serve a technological purpose in food, including emulsifiers (prevents food separation), antioxidants (delays food spoilage caused by oxidation), and substances used for organoleptic purposes (colors, flavors, sweeteners). This narrower international definition of food additives helps provide context for the purpose of adding substances to food. The US definition of food additive is broader than that of Europe and Codex, as it not only includes substances added for a technological function (the international definition of food additive) but also substances added for nutritive purposes (that are not considered food additives in the European Union or under the Codex standard). For example, under the US definition of food additive, the addition of vitamin D or folic acid to food (as a source of essential nutrients) would be classified as a “food additive,” whereas that same addition in the European Union would not be classified as a “food additive”; it would be classified as a nutrient. *Ingredient* is another term that can be used for substances intentionally added to food. Ingredients are listed on food labels, including ingredients added for both technological and nutritive purposes, with some limited labeling exceptions.

Food substances that do not have an established regulatory authorization to be used in food are referred to in some geographies as *novel food*s. Importantly, the term novel food is a regulatory designation, rather than something inherent to the substance itself. A novel food designation for a food substance must consider its specific conditions of use, including both the food category in which it is used and the level at which it is used in that food category. As such, a food substance may not be considered novel under a specific condition of use (e.g., up to 10 mg/kg in bakery products) but novel under other conditions of use (e.g., at 100 mg/kg in bakery products, or at 10 mg/kg in yogurt).

Countries have different regulatory processes established that allow food manufacturers to introduce new/novel food ingredients to food products. In the United States, several regulatory pathways exist including the Food Additive Petition process (21 CFR Part 171), the Color Additive Petition process (21 CFR Part 71), and the Generally Recognized As Safe process (21 CFR Part 170) (U.S. Food and Drug Administration 1977a, 1977c, 1977d). The scientific standard for establishing the safety of food substances is identical across each of these mechanisms: reasonable certainty in the minds of competent scientists that the substance is not harmful under the conditions of its intended use. The determination of safety is a comprehensive, science‐based approach that considers the identity, proposed use, and dietary exposure of a substance to ensure reasonable certainty of no harm under the intended conditions of use.

The mechanisms for gaining authorization for new/novel food substances in other countries have similar requirements for demonstrating the safety of these substances, although the nomenclature for these processes can differ. Importantly, regulations governing novel foods establish that food substances that are substantially similar to those already authorized for use, where any differences do not affect the composition, structure, or nutritional value, are not considered novel. For example, changing the manufacturing process of a food substance in a way that does not significantly alter its characteristics would not be considered a novel food under the novel food regulations in the United States, European Union, and elsewhere the regulatory frameworks of these countries have been adopted. However, emerging manufacturing methods such as precision fermentation have challenged traditional assumptions about ingredient identity, resulting in a debate over how the term “novel” might apply in these cases.

*Processing aids* are a subset of food substances that are used for a technological effect during the production of food but do not have a technological effect in the finished food, such as anti‐foaming agents, clarifying agents, enzyme preparations, and filtration agents. Under regulatory frameworks that incorporate principles of Good Manufacturing Practices (GMPs), the use of processing aids is limited to the minimum amount necessary to produce the technological effect during processing, and these substances are usually only present at negligible levels in the finished product. Similar principles are reflected in the Codex Alimentarious standards, which also incorporate GMP principles to ensure substances are used only at the necessary levels to achieve the intended technological purpose.

Under the US regulation, a processing aid is considered an incidental food additive, but processing aids are not classified as food additives under international food additive definitions. As processing aids are considered incidental food additives found at insignificant levels in finished food products, these are not included in food product labels in the United States, the European Union, or countries that follow Codex standards.

*Food chemical contamination* refers to the unintentional presence of substances in food, which might have the potential to cause illness in consumers. A *contaminant* can be inherent to food ingredients, created during manufacturing processes or resulting from microbiological contamination. Examples of inherent contaminants are elemental impurities such as heavy metals (e.g., lead, cadmium, arsenic), which many regulatory agencies have established regulatory limits for, such as through the US Food and Drug Administration's (FDA) Closer To Zero program (U.S. Food and Drug Administration 2021). Mycotoxins, such as aflatoxins, represent an example of contaminants that occur in raw agricultural commodities due to microbiological activity and for which limits have also been established by regulatory bodies. These contaminant regulatory limits consider the capability of analytical chemistry methodologies that can detect trace levels of contaminants in food matrices, often times at toxicologically insignificant levels. While mitigation approaches may exist for some contaminants, it is not always possible to completely remove them because they are often inherent in the environment or produced during necessary manufacturing processes (e.g., heating or baking).

As contaminants can be found in food but are not considered ingredients, these are not included in food product labels in the United States, the European Union, or countries that follow Codex standards, but these must adhere to the applicable regulatory limits.

In a different context, the terms readily avoidable and not readily avoidable have been proposed for the categorization of substances based on the risk management measures that can be used to control their presence in food. *Readily avoidable substances* are substances intentionally added to food, where manufacturers have control over their presence. As such, risk management strategies can include their reduction or elimination from food (e.g., substitution, new source/supplier, reformulation). Examples of readily avoidable substances include all substances intentionally added to foods including essential nutrients (e.g., vitamins, minerals, macronutrients), other functional ingredients added to provide a benefit to the consumer (e.g., fibers, probiotics), substances added for a technological purpose in the food (e.g., antioxidants, stabilizers), and substances added for organoleptic or sensory purposes (e.g., flavors, sweeteners).

Alternatively, not readily avoidable substances are those present in foods as a result of their transfer from the production environment (e.g., soil, water or air) or their formation during food processing. Reducing the levels of these substances in food can be challenging and often requires extensive mitigation strategies. Examples include naturally occurring environmental contaminants (e.g., heavy metals), naturally occurring toxins (e.g., mycotoxins), and substances formed during food preparation processes (e.g., acrylamide).

Importantly, the terms readily avoidable and not readily avoidable are not descriptions of a consumer's ability to avoid a substance, but rather an indication of the ability of a food manufacturer to prevent their occurrence in a food. For example, consumers could easily avoid not readily avoidable allergens because of mandatory allergen declarations on food labels, despite manufacturers not being able to readily prevent the presence of peanut protein in peanut products.

## 3 Food Safety Risk Analysis

One of the core responsibilities of many toxicologists is *risk analysis*. The Codex Alimentarius Commission has defined risk analysis as “a process consisting of three components: risk assessment, risk management, and risk communication.” Once a risk assessment is conducted, risk management and risk communication can occur. Although all three components (assessment, management, and communication) are important individually, they need to be considered as part of a continuum. To better communicate the risk of chemicals in relative terms, a more nuanced understanding of chemical food safety is needed.

*Food safety* is the assurance that the consumption of a particular food item has reasonable certainty of no harm to humans. Determining that a food is safe does not imply that the product is “free” of contaminants or potential hazards, it implies that exposure to all substances in the food is unlikely to cause an adverse health effect. Food safety determinations are built on science‐based *risk assessments*, the main components of which are hazard identification involving a dose–response assessment, a consumer exposure assessment, and risk characterization based in both hazard and exposure. Risk assessment is a determination of the likelihood of experiencing adverse health effects based on quantitative assessment of both hazard and exposure.

The most commonly confused element during risk analysis is understanding a clear difference between *hazard* (the intrinsic ability of a substance to cause harm) and *risk* (the likelihood that a substance will cause harm), as the latter is often conflated with *hazard*. Stating that something is a hazard is often the highlight in mainstream media when communicating food chemical safety to the public, for example, “chemical X was detected in these three commonly consumed foods.” This communication lacks critical context regarding whether the amount of a chemical detected presents a risk to consumers as risk considers both hazard and exposure. Using hazard‐based terms without qualifiers frustrates risk communication. For example, describing both electricity and processed meats as hazards without the additional context of exposure may distort risk perception by downplaying the high acute risk associated with certain electrical exposures while exaggerating the perceived level of concern of processed meat consumption, which is typically associated with a lower chronic risk.

*Hazard identification* is the process by which potentially harmful substances are assessed for their ability to cause adverse effects in a consumer at escalating doses. Importantly, a hazard assessment does not account for the likelihood of actually observing adverse effects, whereas risk assessments do account for this probability. When conducting a risk assessment, the toxicologist will identify all the available information on the toxic effects of a substance (hazards) and subsequently conduct an *exposure assessment* to quantitate the amount of that substance a consumer would be exposed to. The combined assessments of both hazard and exposure allows toxicologist to determine the possible risk associated with exposure to a given substance. In our food, we rarely consume a single substance, even fruits and vegetables are a complex mixture, which can add to the complexity of risk assessments.

One of the best known examples of a hazard classification process is the one employed by the International Agency for Research on Cancer (IARC). The IARC approach for classifying substances as carcinogenic employs a strength of the evidence (rather than a weight of evidence approach) to define substances in one of four categories (Samet et al. 2020). The objective of the IARC classification is not to draw a conclusion about the risk posed to consumers, as it is only a hazard classification. This is well exemplified in a 2023 statement co‐released by the IARC and the Joint Expert Committee on Food Additives (JECFA). In this statement, the two agencies explain their respective assessments on aspartame, highlighting that the IARC assessment is a hazard classification, while the JECFA conducted a risk assessment that takes the additional step of determining the potential impact to consumers based on both hazard and exposure (World Health Organization 2023).

There are multiple models that can be implemented to estimate exposure to a particular substance in food. The *Estimated Daily Intake (EDI)* is an estimate of the total amount of a substance in food that a consumer is likely to consume in 1 day. It is calculated as the product of two factors: occurrence, the concentration of the substance within food; and consumption, the amount of these foods that a consumer eats. Depending on the needs of the risk assessment, different estimates of intake can be used. Often an average EDI is calculated, and depending on the needs of the risk assessment, a 90th, 95th, or 97.5th percentile estimate of intake could be used. There are different methods available to estimate the total daily intake of food substances, most notably deterministic and probabilistic models.

*Deterministic models of exposure* use a point estimate for both occurrence and consumption to estimate exposure. Compared to other methods, deterministic estimates of exposure require fewer resources to conduct, and while less refined, can provide a conservative estimate of exposure for risk assessments. Deterministic models of exposure are recommended as a screening tool (World Health Organization 2009), and if the model identifies that exposure could present a risk to consumers, a probabilistic model would be warranted to refine the estimates of exposure.

*Probabilistic models of exposure* require the use of an expanded dataset to increase the accuracy of estimates. Instead of expressing the occurrence and consumption of a food substance in point estimates, a probabilistic model accounts for the reported ranges of these values. For occurrence, this means that instead of assuming every sample contains the same concentration of a food substance (e.g., the mean or 90th percentile), the concentration used to calculate exposure reflects the distribution of concentrations seen in food. Similarly, for consumption instead of assuming every individual consumes the same amount of food every day, a range of consumption rates are used to reflect the quantity of foods that are typically consumed.

Probabilistic modeling can be especially useful for risk assessments where there is a broader distribution of occurrence (e.g., concentrations of a naturally occurring substance, such as a mycotoxin) or consumption (including potential consideration of multiple foods over multiple days). In cases where the distribution of a substance is relatively constant (e.g., addition of a food additive for a technological purpose) and consumption is relatively constant (e.g., consumption of infant formula), probabilistic modeling and deterministic modeling may return similar results. Both approaches have applications that they are better suited for, but as with other models, it is also important to be aware of the uncertainties that are inherent in these models (Kettler et al. 2015). Historically, many food substance exposure assessments have relied on deterministic approaches. However, opportunities exist to incorporate more probabilistic approaches in order to refine the exposure assessments that are being used for risk assessment (Chiu and Paoli 2021; Paoli et al. 2025).

The fundamental principles for the toxicological risk assessment of food substances are equivalent to those used when assessing the risk of chemicals in other fields (e.g., pharmaceutical, agrochemical). While some of the particular methods may be different, such as how exposure is calculated, the same scientific principles still apply. Having a strong foundational basis for food safety risk analysis that is common across multiple fields provides additional confidence in the outcomes of food safety risk assessments. However, driving the harmonization of language across the food toxicology field is essential to communicating the outcomes of food safety risk assessments more accurately, consistently, transparently, and effectively.

## 4 Risk Assessment Versus Risk Management

**FIGURE 1:**
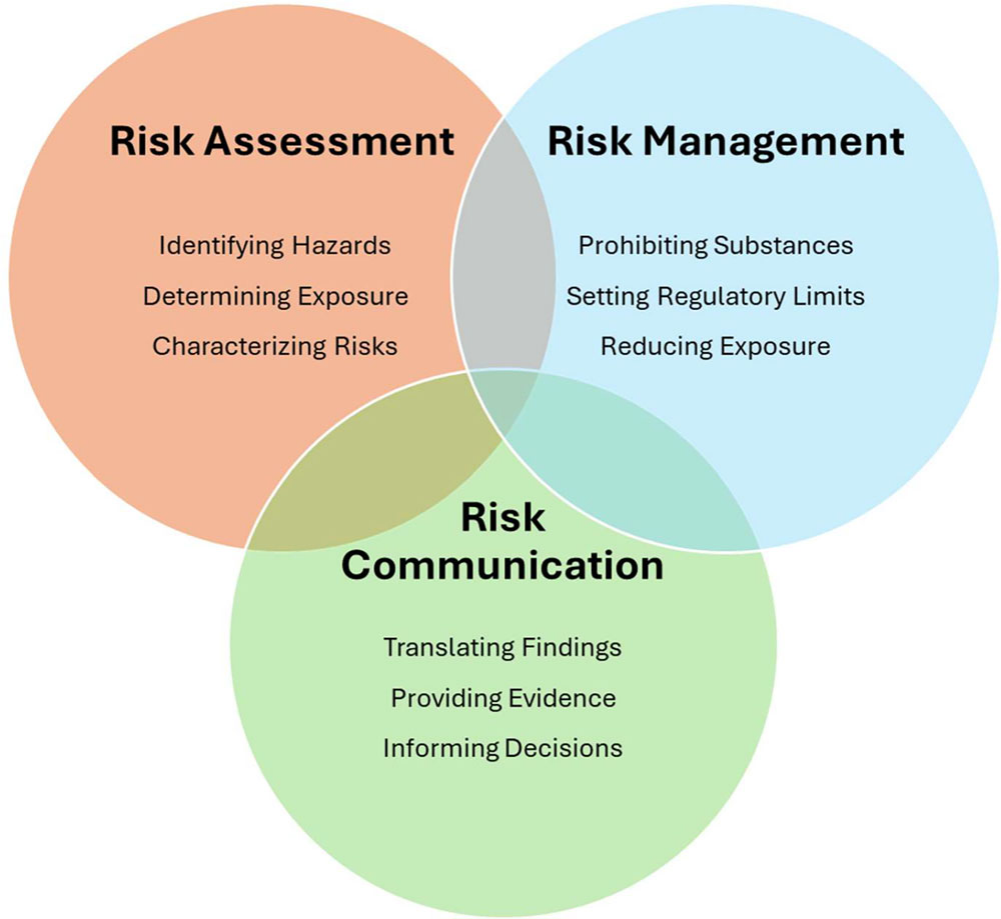
The three core components of risk analysis: risk assessment, risk management, and risk communication. Risk assessment provides the scientific evaluation of hazards, determines exposure, and characterizes risk. Risk management uses this information to establish regulatory actions aimed at controlling exposure to a substance and/or the occurrence of such substance. Risk communication integrates the perspectives of risk assessors and risk managers to facilitate clear and transparent communication of risk findings and associated recommendations to the public and stakeholders, supporting informed decision‐making.

As described above, a *risk assessment* is a science‐based evaluation of data with the objective of determining “is there a human health concern from the level of exposure to this food substance,” whereas *risk management* has the objective of determining “what are we going to do about this food substance.” As such, risk management is the identification of practical steps that can be taken to reduce potential consumer exposure (and therefore risk) from a substance (National Research Council [NRC] 2009). *Risk communication* is the exchange of information and opinions among risk assessors and risk managers to inform the public and stakeholders about the significance of risks and recommended risk controls. Collectively, these components establish a framework that guides how food safety risks are managed in real‐world scenarios (Figure 1).

Food safety risk management manifests in many ways depending on which substance is the subject of the risk assessment. Risk management steps may include prohibiting the intentional addition of substances to food (e.g., partially hydrogenated oils), setting limits on the concentrations of contaminants in food (e.g., FDA Closer to Zero Action Levels for heavy metals), providing guidance on how food manufacturers can reduce occurrence (e.g., FDA Guidance for Industry on Acrylamide in Food 2016), and/or providing consumers with advice on how they can reduce exposure to the substance (e.g., FDA Advice about Eating Fish 2024). Effective risk communication is incorporated within each of these actions to help translate technical risk information into practical guidance that facilitates informed decision‐making by industry and consumers. Broader contextual factors, including economic feasibility, consumer preferences, cultural practices, and historical patterns of use, may add further complexity to the development and implementation of risk management measures.

The risk assessment and risk management processes employed by the United States, European Union, and Codex Alimentarius are structured very similarly. Their approach begins with a scientific risk assessment conducted by risk assessment bodies: the FDA in the United States, the EFSA in the European Union, and the JECFA at Codex. These risk assessment agencies conduct risk assessments using established risk assessment principles. Once the risk assessment is complete, the risk management authority uses it to establish appropriate risk management measures. In the United States, the risk management and risk assessment functions are carried out by different entities within the regulatory framework of the FDA, whereas a separate organization serves as the risk management authority in the European Union (the European Commission) and Codex Alimentarius (the Codex Committee on Contaminants in Foods and Food Additives).

Regulatory limits are common risk management actions for certain substances found in food. The term maximum limit (ML) defines the highest concentration of a contaminant (e.g., heavy metals, mycotoxins) or food ingredient that is considered to be compliant with the relevant regulatory authority. For pesticides and veterinary drugs residues, this limit is referred to as the maximum residue limit (MRL), and it is based on good agricultural practices and safety assessments. In the United States, these residue limits are known as tolerances. Both the ML and MRL can differ between food product categories and countries of product manufacturing and export. The risk management actions taken by food manufacturers to meet maximum levels for readily avoidable substances are typically straightforward, as these substances can be managed through product formulation or through acceptance criteria. The risk management actions for not readily avoidable substances can be much more complex.

Regulatory limits for not readily avoidable substances have been established by the European Union (Commission Regulation (EU) 2023/915) and Codex Alimentarius (Codex Standard 193‑1995), and all or portions of these lists have been adopted by many countries around the world. The United States has also established regulatory limits (action levels) for some not readily avoidable substances and has announced its intention to establish more limits as part of the US FDA Closer to Zero and Post‐Market Assessment programs (U.S. Food and Drug Administration 2021, 2024). The process used by these agencies to establish regulatory limits uses the output of the risk assessment to evaluate the impact on consumer risk, but importantly also incorporates additional considerations such as the capabilities of analytical methods and the technological feasibility of reducing contaminants in specific foods. Differences in the conclusions of the risk assessment and practical constraints on risk management recommendations can both lead to differences in regulatory limits between countries.

At present, there is close alignment of limits for not readily avoidable substances by different regulatory authorities around the world, but deviations are sometimes necessary. For example, there are cases where natural variations in soil composition and contamination, such as differing heavy metal levels in soil, can make it difficult for farmers in a country to meet the limits determined to be appropriate elsewhere. An assessment of cadmium and lead in cocoa products in the US market concluded that the residues of these heavy metals are significantly correlated to the product geographic origin (Abt et al. 2018). This is a good example of where country‐by‐country differences in risk management decisions can exist. Additionally, expert judgement can play a role in establishing regulatory limits due to differences in the identification of critical toxicological endpoints, the selection of a point of departure, and the application of uncertainty factors. This variability arises from the legitimate differences in the scientific interpretation of the whole weight of evidence as expected between international risk assessment and risk management practices.

Ideally, risk management steps are implemented to significantly reduce consumer risk, bringing exposure that had been estimated to exceed a health‐based guidance value to an exposure that would be estimated to be below a health‐based guidance value. While risk management strategies are typically supported by science‐based risk assessments, there are instances were scientific uncertainty or data gaps complicate decision‐making. Beyond the science, risk management is also driven by policy considerations. In the past, this has sometimes led to instances where risk management recommendations are misaligned with the science.

A good example of this is the 2025 revocation of the authorization of FD&C Red No. 3 (also known as erythrosine or INS 127) as a certified food dye by the US FDA (U.S. Food and Drug Administration 2025c). This color additive continues to be permitted in some foods as a color additive in the European Union and Codex based on risk assessments that determined there was no appreciable risk to consumers (European Food Safety Authority 2011; Joint FAO/WHO Expert Committee 2018). In their announcement, the FDA emphasized that this risk management measure (revocation of the color's use) was not due to a safety concern, aligned with the conclusions of other risk assessment agencies. However, in the case of FD&C Red No. 3, the FDA was obligated to revoke its use because of the Delaney Clause, a provision in the US food law that stipulates that no substance could be legally added to food if it was found to cause cancer “in man or animal” (United States Congress 1958). This legal requirement has come under significant scientific scrutiny as our understanding of carcinogenesis has evolved (Weisburger 1996; Felter et al. 2020; Krishan et al. 2021), especially in cases like FD&C Red No. 3 where the scientific evidence clearly demonstrated that the toxicological findings observed in rats were not relevant to humans (Joint FAO/WHO Expert Committee 2018). In these instances, as the whole weight of evidence is not considered, it can result in overconservative regulatory actions.

The *precautionary principle* is another example of a risk management policy, which employs a strictly hazard‐based approach to restrict usage or exposure to a particular chemical. The precautionary principle was originally established for environmental purposes but has since been applied to other sectors including food. The Precautionary Principle recommends actions to reduce exposure to food substances even when there is scientific uncertainty as to whether those actions would have meaningful benefits for reducing consumer risk. Its application in food policy decision‐making has been controversial (Martuzzi and Bertollini 2004; Martuzzi 2007; Mepham 2011; Lofstedt 2014; Herman and Raybould 2014; Miller and Conko 2018; Tagliabue 2018), with advocates noting that its basis is the protection of consumer health and prevention of uncertainty from delaying action. Those critical of the principle note that it recommends arbitrary, hazard‐based decisions rather than risk‐based decisions founded on existing scientific evidence.

For substances without an established safety threshold, the European Union has historically employed the as low as reasonably achievable (ALARA) precautionary approach to guide risk management decisions. In this context, risk management measures are generally aimed at achieving an appropriate level of protection (ALOP), defined by the World Trade Organization as the level of protection a country considers appropriate, often expressed as the acceptable level of risk to protect human health within its territory. More refined approaches such as the margin of exposure (MOE) and the threshold of toxicological concern (TTC) have been developed and implemented by risk assessors to establish frameworks that support prioritization actions based on the exposure and toxicological profile of a substance.

## 5 Toxicological Approaches

The methods used to generate the scientific data to support food safety risk assessment have evolved significantly since the advent of modern toxicology practice and will continue to evolve to incorporate newer and human‐relevant approaches. In 1902, Harvey Wiley created the Poison Squad as the first model for generating safety data on food substances to support risk assessment and risk management decisions (Lewis 2002). The Poison Squad was a group of human volunteers that consumed increasing amounts of food substances, and Dr. Wiley and his fellow researchers would note when the amount of those food substances caused adverse effects (Lewis 2002). While the relevance of a human model is difficult to refute, it presents ethical challenges, and it is not a sustainable model for conducting food safety studies. Since that time, many new approaches have been developed.

The first evolution of food safety studies was the establishment of *traditional safety studies*. Today, the suite of traditional safety studies includes in vivo animal models that characterize the short‐term (acute) and long‐term (sub‐chronic and chronic) effects of repeated ingestion of a substance, as well as in vitro assays to assess specific endpoints such as genotoxicity (European Food Safety Authority 2021). While all available safety studies must be considered part of a risk assessment, more emphasis should be given to studies that have been validated for the outcomes they are measuring. Validated studies, such as those established by the Organisation for Economic Co‐operation and Development (OECD), establish strict criteria on how a study must be conducted to ensure the accuracy and reproducibility of the results, providing confidence that results generated using that method are causal and can have relevance to human health (and thus risk assessment). A breadth of validated studies exists, providing opportunities to conduct evaluations of specific endpoints (e.g., immune function, reproductive and developmental outcomes) that may be needed, depending on the specific substance and the population assessed (Rahmani et al. 2024). Although traditional safety studies are considered in most cases the gold standard for the purpose of risk assessments, scientific advancements are introducing new methodologies to assess the safety of food substances (Wood et al. 2025).

The evolution of science and a desire to find more human‐relevant methods has led to the creation of another type of safety studies referred to as new approach methodologies (NAMs). NAMs are alternative methods to assess safety and may include in vitro, in silico, and in chemico approaches, or an integration of multiple approaches (i.e., integrated approaches to testing and assessment) that help reduce the use of animals in safety testing. There can be some disagreement on what types of studies would be considered NAMs, but the broad definition presented here incorporates some traditional safety studies (even though they are not “new”), such as the Ames assay and in vitro micronucleus assay because they do not involve the use of animals.

The United States has been increasing efforts to develop NAMs since 2007, when the NRC published “Toxicity Testing in the 21st Century: A Vision and a Strategy.” This report provided a framework for toxicity testing in the 21st century which focused on a more efficient, predictive, and economical system for assessing the effects of chemicals (Gibb 2008). Since then, there has been an explosion of research in this area with strategies for implementation of NAMs into risk assessment being published by several US agencies including the FDA. In the European Union, there has also been an effort to reduce animal use and instead adopt more NAMs methodologies such as the EU directive 2010/63/EU, which stipulates the legal obligations to replace, reduce, and refine the use of animals in scientific procedures (European Parliament and Council 2010).

One of the NAMs that has been used to generate data is the leveraging of high‐throughput screening assays. This approach uses in vitro methods in a way that enables rapid screening of thousands of substances for specific endpoints and has been integrated into efforts such as its *Predictive Toxicology Roadmap* in 2017 (Fitzpatrick 2020). There are many challenges in understanding their applicability for different types of substances, and global regulatory acceptance of these methods. Toxicology in the 21st Century, a federal collaboration among US Environmental Protection Agency (EPA), National Institutes of Health (NIH), and the US FDA, has allowed the generation of huge amounts of NAMs data, but questions remain as to how to incorporate these results into risk assessment and risk management activities (Tox21 Consortium 2008; Punt et al. 2020). In fact, the ability of foods themselves to activate many of these assays raises serious concerns about how to interpret these results in the context of a complicated food matrix (Wetmore et al. 2019). As described above, the most valuable studies for risk assessment are those that have undergone validation, such that they provide the maximum confidence regarding the relevance of results to human health.

When data gaps are identified, *read‐across* is an approach that can be implemented to fill gaps by using available information from structurally or mechanistically similar substances. This approach is based on the principle that substances with similar chemical structures and/or properties are likely to present similar toxicological effects and implications. Read‐across approaches may incorporate multiple sources of evidence, including experimental data, physicochemical properties, and computational tools. One example of these supporting tools is *Quantitative Structure–Activity Relationship (QSAR)*, which predicts the activity of a molecule based on its chemical and physical properties. This is achieved using a mathematical model that correlates the molecular structure with the activity of similar chemicals with known effects. Read‐across can also be supported by other tools such as the TTC. This approach uses large datasets with hundreds of substances with known toxicological profiles to determine across substances with similar chemical characteristics what is the lowest concentration that has been shown to produce an adverse effect (Boobis et al. 2017). By establishing a conservative *threshold* for these classes of compounds, risk assessors can be confident that other substances with similar chemical characteristics, for which no toxicological data exist, will also not produce adverse effects if exposure is below that threshold. This scientifically established concept was originally implemented with the Cramer classifications (Kroes et al. 2004) and has recently been updated by the FDA with their publication of the expanded decision tree (EDT) framework (U.S. Food and Drug Administration 2025b).

While the TTC is often used for prioritization based on the exposure and toxicological profile of a substance, it has also shown to be a useful tool for supporting risk assessment and is part of the CODEX guidelines for rapid risk analysis (Codex Alimentarius Commision 2019). The TTC has been leveraged for the evaluation of constituents in botanicals, low‐level impurities in drinking water, pesticide metabolites, and low‐level containments present in food (Canady et al. 2013; Mahony et al. 2020). Another example of the practical implementation of the TTC is in the review of flavoring substances (Kroes et al. 2004). Since intakes of flavor substances are extremely low and are self‐limiting in taste, exposures to these substances are typically well below the thresholds established by the Cramer Classifications or EDT.

There are many efforts to increase the use of NAMs, including in silico methods such as Cramer Classification and the EDT. The US EPA had announced plans to phase out animal testing by 2035, but this deadline was then abandoned to ensure that its implementation is science driven. The FDA made a similar announcement in April 2025, although it did not specify a target year. Prior to it becoming mandatory, scientists across multiple disciplines have been exploring opportunities to incorporate more NAMs into food safety risk assessment (Parish et al. 2020; Schmeisser et al. 2023). Recent analysis has shown that there is the potential to incorporate more NAMs into risk assessment today (Wood et al. 2025), but at the same time, scientific and policy challenges remain before there can be a full replacement of traditional toxicology methods.

Human evidence can also play a role in food safety risk assessment. For substances such as environmental contaminants, there are ethical limitations on the ability to conduct prospective human intervention studies. In these cases, *epidemiological studies* serve as the only method available to integrate human evidence into a risk assessment. While there are certainly benefits from having data in humans (rather than an animal, in vitro, or in silico model), there are many methodological challenges regarding epidemiological studies that limit their utility for food safety risk assessment (Christensen et al. 2015; Déglin et al. 2021). These challenges include difficulties in accurately estimating exposure, identifying appropriate control populations, and ensuring the availability and reliability of outcome data, as well as the fundamental limitation that epidemiological studies cannot establish causation on their own and therefore their findings reflect correlation rather than causation. As such, while epidemiological data may help support food safety risk assessment, they are often considered supportive or confirmatory rather than conclusive in the absence of complementary evidence from additional study designs.

*Clinical studies* can be a mechanism for evaluating human effects of a substance, particularly for substances such as nutrients and food additives that are intentionally added to foods. Unlike epidemiological studies, clinical studies can be designed in a manner that allows causative determinations. These studies can be designed with a controlled dosing paradigm to evaluate the relationship between the dose of a substance and an outcome. Additionally, targeted clinical studies can evaluate whether a mechanism of toxicity seen in an animal or cell culture study is relevant to humans. Clinical studies do have some limitations compared to non‐human studies. These include that it is quite difficult to control study conditions during a human study (as opposed to animal studies that are conducted under highly controlled conditions) and that there is a limitation in the endpoints that can be measured (animal studies typically include full microscopic and macroscopic inspection of tissues that is of course not possible in a human study).

Two examples where clinical data were used to support risk assessment are the evaluations of FD&C Red No. 3 and perchlorate. A human study was used to demonstrate that the thyroid disruption caused in rats by FD&C Red No. 3 was not relevant in humans, which allowed risk assessors at the FDA, EFSA, and JECFA to conclude that this mechanism that was responsible for thyroid tumors in rats was not relevant to humans (European Food Safety Authority 2011; Joint FAO/WHO Expert Committee 2018; U.S. Food and Drug Administration 2025c). Similarly, as limitations are known to exist in using rats as a model for human thyroid function, a clinical study evaluating dose response in humans serves as the pivotal study in determining the health‐based guidance value (HBGV) for perchlorate (Haber et al. 2021; European Food Safety Authority 2025).

While examples do exist where human clinical data have supported or even driven risk assessment of food substances, it is important to note that the majority of food safety risk assessments rely on results from non‐human studies as the pivotal data. This reflects practical and ethical constraints on clinical studies for food additives, including limitations on achievable exposure levels, challenges controlling background dietary exposure, and the complexity of designing studies with sufficient duration and sensitivity to detect chronic or low‐incidence effects. This results in clinical data being used to inform the human relevance for specific endpoints rather than serving as the primary basis for hazard identification. The utility of preclinical studies for food risk assessment also reflects the success these validated models have had in evaluating the safety of food substances, but also other sectors such as pharmaceuticals or agrochemicals.

A food safety risk assessment must consider the totality of the evidence, and there are different approaches and tools that can support such an assessment. The best practice in food safety risk assessment is implementing a *Weight of Evidence* approach in which a hypothesis is tested against the best information available. A weight of evidence approach can integrate data from multiple types of studies and allows for weighting the results of those studies differently to emphasize studies that had the best study design or the most human relevance. A *systematic review* is a tool that can support a weight of evidence evaluation (Suter et al. 2020). Systematic reviews have been instrumental in supporting risk assessment of both readily avoidable substances (Wikoff et al. 2017) and not readily avoidable substances (Fitch et al. 2024).

While sounding similar, a *strength of evidence* review differs from a weight of evidence review in key aspects. A strength of evidence review begins with a hypothesis and then evaluates the strength of the data that support this hypothesis, without considering studies that may contradict that hypothesis and then weighing the strength of those studies against each other. The IARC is one agency that utilizes a strength of evidence approach to support their hazard classification system, which is a factor in how their classification of substances as carcinogens is at times at odds with risk assessments that take a weight of evidence approach.

Many factors can reduce the translatability or relevance of toxicological findings to humans. These include unrealistic exposure scenarios, biological differences between humans and animal models, and species‐specific molecular pathways. One tool that has been useful for assessing these factors is the *Bradford Hill criteria*, nine principles that can be applied to determine whether an observed effect following exposure to a substance is causal or just associative (Hill 1965). Applying a structured evaluation with a tool like the Bradford Hill principles strengthens conclusions regarding whether a causal relationship between a substance and observed adverse effect exists by providing a comprehensive, multidisciplinary evaluation of the available evidence, integrating findings from traditional safety studies, NAMS, epidemiology, history of safe use and/or clinical research.

## 6 Other Terms Relevant to Food Toxicology

Food toxicologists and risk assessors use terminology that is shared with other scientific and public health fields. Providing clear definitions within the context of food safety risk assessment can improve understanding, thereby improving consumer communication and increasing transparency in food safety discussions. Some of these terms are as follows:

- Health‐based guidance value (HBGV) is a term used to describe “the safe” amount of a substance that can be consumed over a lifetime without posing a significant health risk to the consumer. A HBGV is the output of a risk assessment and is often derived from safety endpoints (e.g., NOAELs and BMDs) adjusted with the appropriate uncertainty factors (World Health Organization 2009). The HBGV defines an amount of a substance “unlikely to pose a significant health risk,” and this definition of “safe” is equivalent to the “reasonable certainty of no harm in the minds of competent scientists” used by the FDA. HBGVs can be established for both readily avoidable and not readily avoidable food substances. Over the past decades, several varieties of HBGV have been established, including:
- Acceptable daily intake (ADI) is a term used for the estimated amount of a substance that can be ingested daily over a lifetime with no appreciable health risk for the general population (Herrman and Younes 1999; Elsevier 2022). The ADI is used commonly to describe safe levels of intake. For food ingredients, often the output of the risk assessment is the determination of an ADI for the substance (Benford 2000).
- ADI “not specified” is a term used when it is determined that it is not necessary to establish a numerical ADI. This occurs when the available safety data, the total daily intake, and the intended use of the substance are determined to not pose a risk to consumers (Joint FAO/WHO Expert Committee 1995). In these cases, the exposure level is very low relative to the threshold at which adverse effects would be expected to occur. As human exposure is far below level of toxicological concern, conducting animal testing to derive an ADI is scientifically unjustified and serves no practical purpose.
- Tolerable daily intake (TDI) is an estimate of the quantity of a substance that can be found in food and ingested daily over a lifetime without posing a significant health risk (Herrman and Younes 1999; Elsevier 2015). Generally, TDI is the term used for HBGVs established for not readily avoidable substances, while ADI is the term used for HBGVs established for readily avoidable substances.
- Provisional tolerable weekly intake (PTWI) estimates the amount of a contaminant in food that can be ingested over time without health risks. This term is often used to describe contaminants that may accumulate in the body, and the “weekly” designation is used to emphasize the importance of limiting intake over a period of time (Herrman and Younes 1999).
- Reference dose (RfD) is a term primarily used by the US EPA; however, it is occasionally used for food safety risk assessment. The EPA defines the RfD as “an estimate of daily exposure to the human population (including sensitive subgroups) that is likely to be without an appreciable risk of deleterious effects during a lifetime” (U.S. Environmental Protection Agency 1993). This term has the same meaning as the other food‐related terms in this list (e.g., ADI, TDI), but it should be noted that in general applying EPA approaches that are developed for the assessment of chemical pesticides, industrial chemicals, and contaminants found in drinking water and the air is not best practice for food, and in some cases, this US EPA specific endpoint may actually diverge from the ADI determined by other agencies.
- Acute reference dose (ARfD) is the maximum single day exposure, which is anticipated to be without appreciable risk for the general population. Acute toxicity due to substances found in food is generally uncommon so this is not a term often used in food safety risk assessments. However, there are examples where it has been applied for some food‐relevant substances, such as some pesticides and veterinary drugs residues where higher short‐term exposure may occur. It should be noted that food safety risk assessment focuses on both short‐term and long‐term effects of the food substance, and in nearly all cases (food allergens is an exception), the dose required to produce an acute response will be significantly higher than that required to produce a chronic response. Therefore, if risk management measures are being taken to prevent exposure at the lower doses required to produce a chronic response, these risk management approaches would already be addressing other potential acute effects. This is exemplified in the recent EFSA opinion on perchlorate in which this point is made, and it is determined that an ARfD is not necessary (European Food Safety Authority 2025).
- Upper tolerable intake limit (UL) is the highest level of daily nutrient (e.g., vitamins and minerals) intake that is likely to pose no adverse health effects (Yates et al. 2017; National Institutes of Health 2026). This is a term that is used only for nutrients, not other types of food substances. As consumers become interested in the benefits of bioactive food components from plant sources, there is a growing need to establish upper tolerable intake limits for these substances (Yates et al. 2017).
- Maximum allowable dose level (MADL) is a level of exposure at which there is no observable reproductive effect, assuming exposure at one thousand times that level (Office of Environmental Health Hazard Assessment [OEHHA] 2023). It is determined by dividing the no observable adverse effect level by a safety factor of 1000. The California OEHHA uses this unique approach to establish “safe harbors” for chemicals to aid businesses in complying with California's Proposition 65 (OEHHA 2023). The MADL derivation approach is not aligned with other risk assessment approaches (e.g., ADI, TDI) and has not been adopted outside of California.
- Margin of safety (MOS) is the ratio of the NOAEL derived from toxicological studies to the estimated human exposure level (U.S. Environmental Protection Agency 2011). It is a metric to determine if human exposure is sufficiently below the level at which no adverse effects are observed. Typically, an MOS equal to or greater than 100 is indicative of an adequate level of protection for human health. When values are below this threshold, further refinement of the exposure assessment or additional risk management considerations may be required. This tool provides a comparison of observed effect levels and estimated human exposure to support risk characterization of substances that are known to exhibit threshold effects.
- MOE is the ratio of the point of departure (POD) obtained from toxicological studies to the estimated human exposure level (European Food Safety Authority 2012b). The POD is typically a study NOAEL or other relevant dose metric selected for risk assessment. It should be noted that this approach differs from the HBGV in that it does not derive a safe amount of a substance that can be ingested daily over a lifetime with no appreciable health risk. Rather, it is a metric to support risk characterization at different estimated exposure levels. The EFSA Scientific Committee states that “an MOE of 10,000 or higher, if it is based on an animal study, would be of low concern from a public health point of view and might reasonably be considered a low priority for risk management actions”(European Food Safety Authority 2005). EFSA has applied the MOE approach to assess the safety of genotoxic/carcinogenic contaminants and complex substances in food, including those where establishing an HBGV is not feasible (e.g., smoke flavoring) (European Food Safety Authority 2012b). It is important to note that an acceptable margin of exposure in many international jurisdictions for substances found in foods is often 100 and does not need to reach values such as 10,000 to meet the expectation of a reasonable certainty of no harm.

**FIGURE 2:**
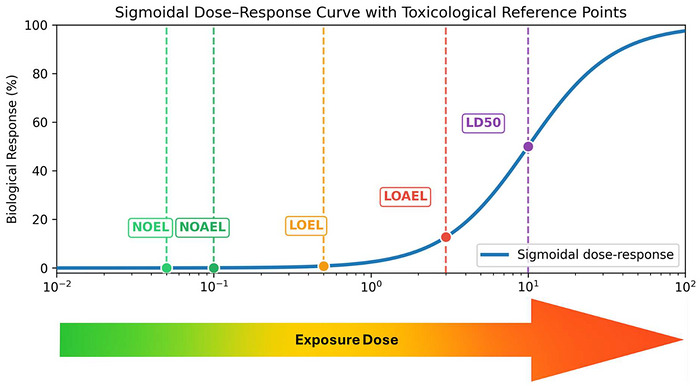
The relationship between increasing the exposure dose (*x*‐axis) and the biological response (*y*‐axis) is represented here by a hypothetical sigmoidal dose–response curve. The main toxicological reference points are plotted along the curve: The no observable effect level (NOEL) and the no observable adverse effect level (NOAEL), respectively, denote the highest dose at which no effect or no adverse effects are measurable. The lowest observable effect level (LOEL) points at the lowest dose at which any effects are detected, while the lowest observable adverse effect level (LOAEL) is indicative of the lowest dose at which an adverse effect is observed; The median lethal dose (LD_50_) identifies the dose that can potentially cause lethality in 50% of the test subjects. Together, these reference points contextualize Paracelsus' principle of “the dose makes the poison” as it illustrates the transition from no observable adverse effects to lethality as exposure dose increases (arrow).

The risk assessment process of deriving an HBGV includes the selection of the most sensitive, relevant endpoint from a safety study to serve as the POD. The POD is the dose administered in a study that produced low or no effect in the model (Sand et al. 2016). There are several outcomes from studies that can serve as a POD for deriving an HBGV, and many terms are used to describe the determination of a POD including:

- Benchmark dose/benchmark dose level (BMD/BMDL) is a state‐of‐the‐art approach to deriving a POD (Haber et al. 2018). A key benefit of this approach is it leverages the responses at doses administered in the study and uses mathematical modeling to extrapolate the likely response at doses that were not tested. This contrasts with the NOAEL/NOEL and lowest observed adverse effect level/lowest observed effect level (LOAEL/LOEL) approaches where the POD *must* be identified from an administered dose, whereas it is not possible to administer an infinite number of doses in a study. This approach is becoming more common for POD derivation, with EFSA publishing a guidance on its use (European Food Safety Authority 2022) and it being applied as part of the risk assessment for 3‐MCPD conducted by the EFSA and the JECFA (Joint FAO/WHO Expert Committee on Food Additives 2016; European Food Safety Authority 2018), as well as other EFSA evaluations such as those for nickel and perchlorate (European Food Safety Authority 2020b, 2025).
- Dose‐response curves are a useful tool in the risk assessment as they depict the relationship between the dose and adverse effects in humans, representing a graphical representation of Parcelsus's statement “the dose makes the poison.” This graph is prepared by plotting dose levels administered in a study along the *x*‐axis, and the measured effect along the *y*‐axis (Figure 2). The most common response in a toxicology study is *not* a linear response across all doses administered, but rather a sigmoidal (S shaped) curve. This sigmoidal shape reflects that a threshold can be established below which no toxicity is seen (flattening the curve at the lowest doses), as the dose increases there is a range with a rather linear response, and at the highest doses, the curve again flattens as there is a dose above which the response no longer increases with increasing dose.
- No observed adverse effect level/no observed effect level (NOAEL/NOEL) is a value derived from a study or calculated from a dose response curve representing the highest dose at which either no *adverse* effects (NOAEL) or no effects *at all* (NOEL) are identified (Food and Drug Administration 2005; U.S. Environmental Protection Agency 2025). In a hypothetical example where doses of a substance of 1, 30, 100, 300, and 1000 mg/kg body weight per day (mg/kg bw/day) were administered, if no treatment‐related adverse effects are identified at 1 and 30 mg/kg bw/day, but adverse effects are observed at 100 mg/kg bw/day (e.g., increase in a set of biomarkers indicating liver toxicity), 30 mg/kg bw/day would be considered both the NOEL and NOAEL. If it was the case that minor‐treatment related, non‐adverse effects (e.g., slight differences in food consumption) were observed at 30 mg/kg bw/day, this dose would remain the NOAEL, but the NOEL would instead be 1 mg/kg bw/day.
- LOAEL/LOEL is a value calculated from a dose response curve representing the lowest dose at which *adverse* effects (LOAEL) or any effects *at all* (LOEL) are observed (European Food Safety Authority 2005; U.S. Environmental Protection Agency 2025). In this same hypothetical example, 100 mg/kg would be considered the LOAEL if the response being measured is considered adverse and the LOEL if the response being measured is not considered adverse (e.g., slight differences in food consumption). Often the reason an LOAEL/LOEL is used if the study did not have a dose where no effect was observed, or potentially if there is a physiological reason to consider the lowest amount of response as the relevant POD.
- Median lethal‐dose (LD_50_) is the dose of a test substance that has been determined to be lethal for 50% of the animal subjects within a test group of a toxicology study (U.S. Food and Drug Administration 2007). The LD_50_ can be useful to compare the hazard potency of chemicals and help characterize their acute toxicological profile. This term is not typically applied in food safety risk assessments as it reflects short‐term toxicity, and it is not appropriate for evaluating the chronic low‐level exposures typically associated with food. Furthermore, LD_50_ testing is being phased out in some jurisdictions, like the European Union which has implemented restrictions for this test as part of a broader transition plan toward the adoption of NAMs.
- Uncertainty factors/safety factors are used in risk assessment to adjust for unknown/incalculable variation in calculating a HBGV from a POD (World Health Organization 2009). These safety factors often include allowances for differences between individual animals as well as differences between laboratory animals and humans (European Food Safety Authority 2012a). Most toxicological data are derived using animal models; therefore, extrapolations accounting for *interspecies* (accounting for variability between different species, e.g., from rat to human) and *intraspecies* (accounting for variability within the same species, e.g., from the most sensitive to the least sensitive human) are often applied. In the 1950s, Lehman and Fitzhugh (1954) proposed a standard 100‐fold uncertainty factor that was then adopted by the FDA in the Code of Federal Regulation (U.S. Food and Drug Administration 1977e). This factor reflects a default 10‐fold allowance for interspecies variability and another 10‐fold safety factor for intraspecies variability (U.S. Environmental Protection Agency 1993). While the 100‐fold uncertainty factor is still often used, risk assessors also adjust these factors to reflect when scientific evidence or lack of sufficient data indicates that either more or less uncertainty exists for an endpoint. The EFSA Scientific Committee has established a guidance to harmonize rather than strictly standardize the use of default values and uncertainty factors in risk assessment, providing a scientific rationale for their application (European Food Safety Authority 2012a).

Another concept relevant for food safety risk assessment is *absolute risk* versus *relative risk*. These are two approaches to quantitating and communicating the outcomes from risk assessment. Absolute risk describes the quantitative probability of an outcome. For example, it is expected for 38.9% of men and women to be diagnosed with cancer over their lifetimes. Relative risk provides a quantitation of the probability of that occurrence in comparison with one or more alternatives (Hazra and Gogtay 2017). For example, in the United States, the absolute risk of dying from a work‐related ladder fall is approximately 9 per 10,000,000 people annually, while the WHO estimates the absolute risk of dying by accidental drowning is 610 per 10,000,000 people annually (Centers for Disease Control and Prevention 2014; World Health Organization 2024). Though these estimates are subject to uncertainty factors related to differences in data sources, reported incidences, and population differences, they suggest that death by drowning is approximately 68‐fold more likely to occur than death by falling off a ladder.

Communicating the outcome of a risk assessment by either absolute or relative risk can shape both risk management decisions and the public perception of an issue. As an example, in a risk assessment of total aflatoxins in peanuts, the EFSA estimated that if the regulatory limit for aflatoxins in peanuts was changed from 4 to 10 mg/kg, the absolute lifetime cancer risk to hepatitis B‐negative individuals would rise from 5 in 10,000,000 to 7 in 10,000,000 (European Food Safety Authority 2020a). This calculates to an estimated relative increase of lifetime cancer risk of 1.6‐fold. The difference in communicating this as absolute risk “your lifetime cancer risk from aflatoxins in peanuts goes from *5 to 7 in 10,000,000*” versus relative risk “you have a *nearly 2‐fold increase* in your lifetime cancer risk from aflatoxins in peanuts” are likely to be perceived very differently by risk managers and consumers.

Estimation of exposure is a critical component of risk assessment, and as described above, having appropriate analytical methods available to quantitate the amount of substances in food is key to accurately estimating consumer exposure. Therefore, having a basic understanding of some of the terminology used when describing analytical methods is crucial to estimating exposure and being able to describe the limitations of these estimates. Some key terms related to analytical methods are as follows:

- *Limit of detection* (LOD) is defined as the lowest concentration of a substance that can be identified using analytical methods, but the concentration detected is too low for it to be quantified with certainty (Elsevier 2013a).
- *Limit of quantification* (LOQ) is the lowest concentration of a substance that can be detected using analytical methods, and for which its concentration can be quantified with certainty (Elsevier 2013b).

*Upper bound (UB), medium bound (MB), lower bound (LB)* are terms to describe how data are managed when the amount of a substance is below the LOQ, and therefore not quantifiable. In an UB approach, values below the LOQ are assumed to equal the LOQ (e.g., if the LOQ is 10, results below the LOQ are assumed to be 10). In an MB approach, values below the LOQ are assumed to be half the LOQ (e.g., if the LOQ is 10, results below the LOQ are assumed to be 5). In an LB approach, values below the LOQ are assumed to be zero. All three of these approaches have utility in risk assessment as they provide mechanisms to have more (UB) to less (LB) conservative approaches to estimating occurrence of a substance. As part of the risk analysis process, it is critical to be transparent in which approach(s) are taken to provide the appropriate context for risk managers.

## 7 Conclusion

Food safety risk assessment has undergone incredible improvements since 1902 when Harvey Wiley tested the safety of food additives on volunteers at the US Department of Agriculture, yet the principle first expressed in the 16th century by Paracelsus still makes up the foundation of this science. But food safety risk assessment is a science, and as such we should expect it to continue to evolve as it adopts new scientific findings and approaches. Even in an environment where this science will be continually evolving, it is still important to establish a harmonized language that allows for clear and transparent communication.

Risk assessment agencies around the world have adopted highly aligned approaches to food safety risk assessment, as evidenced by the assessments from risk assessors such as the FDA, EFSA, and JECFA coming in most instances to similar conclusions in their scientific risk assessment of both readily avoidable and not readily avoidable substances. This paper also recognizes that even in cases where practices are aligned between agencies, misalignment with the terminology used to describe the food safety risk assessment processes can lead to the perception that there are differences, which in turn can lead to the perception that there are no best practices. This paper attempts to demystify the underlying concepts and principles that serve as the foundation of the approaches used by these agencies.

The late Arnold Lehman, who was integral to establishing the 100‐fold uncertainty factor, highlighted the complexity of toxicology in the mid‐20th century by noting that “You too can be a toxicologist in two easy lessons, each of ten years.” Decades later, toxicology remains a complex and continually evolving field that requires ongoing adaptation by those who practice and communicate it. As such, food safety risk assessment is a highly technical discipline, and the scientific processes underlying risk assessment and risk management decisions are not always readily accessible to consumers. The purpose of this paper is to make these highly technical topics more approachable for scientists and communicators who may interact with consumers, as there is great value in bringing better understanding of these concepts to a broader audience.

In an era where many individuals seek information through social media platforms, they may encounter information of variable quality, including content that is incomplete or misleading. To address this, academic, governmental, and private sectors must collaborate and maintain open and transparent communication with the public, clearly outlining the steps taken to ensure food safety. As consumers become better informed, they are better equipped to make decisions that support their health and nutritional needs. To properly inform consumers, it is crucial to effectively communicate risk assessment findings, yet this is a challenging aspect of the food safety system. As public interest grows around the safety and implications of contaminants in food, it becomes essential to clearly differentiate between hazard and risk, two concepts often misunderstood and miscommunicated. Clear and transparent risk communication helps protect consumers from the influence of misleading content contributing to the fear that deteriorates public trust in the food safety system, highlighting the need to communicate the proactive and scientifically rigorous processes in place to ensure food safety.

This review is intended to serve as a resource on risk assessment best practices, aiming to improve understanding of its components and the distinction between hazard assessment and risk assessment. Clear communication of these concepts can help consumers interpret and evaluate the risks associated with substances in food more accurately—always keeping in mind that *the dose makes the poison*, rather than the erroneous notion that exposure to any amount of a substance should be considered detrimental.

## Author Contributions

**Paul R. Hanlon**: writing – review and editing, conceptualization, writing – original draft, validation. **Kenneth S. Rivera‐González**: conceptualization, writing – original draft, writing – review and editing, validation, visualization. **Jay S. Petrick**: writing – review and editing, conceptualization. **Christine M. Crincoli**: writing – review and editing, conceptualization. **Matthew D. Teegarden**: writing – review and editing, conceptualization.

## Conflicts of Interest

K.R.G., M.T., and P.H. were full‐time employees of Abbott Nutrition during the drafting of this manuscript. C.C. was a full‐time employee from Cargill, Inc. during the drafting of this manuscript; and during their employment, they received stock options. J.P. reports a relationship with Archer Daniels Midland Company (ADM) that includes employment and equity or stocks.
